# Ferulic Acid Alleviates Radiation-Induced Immune Damage by Acting on JAK/STAT Signaling Pathway

**DOI:** 10.3390/ph17091175

**Published:** 2024-09-05

**Authors:** Mingyue Huang, Anping Ye, Haoyu Zhang, Junru Chen, Tingyu Yang, Xue Wei, Yue Gao, Zengchun Ma

**Affiliations:** 1 Department of Pharmaceutical Sciences, Beijing Institute of Radiation Medicine, Beijing 100850, China; 15907489303@163.com (M.H.); anpingye@163.com (A.Y.); zhy16645122363@163.com (H.Z.); 15088094928@163.com (J.C.); 15254555178@163.com (T.Y.); 17861175821@163.com (X.W.); 2 Department of Pharmaceutical Sciences, Jiangxi University of Traditional Chinese Medicine, Nanchang 330006, China; 3 School of Traditional Chinese Medicine, Guangdong Pharmaceutical University, Guangzhou 510006, China; 4 Tianjin University of Traditional Chinese Medicine, Tianjin 300193, China

**Keywords:** aging, ferulic acid, immune system injuries, inflammation, ionizing radiation

## Abstract

The disruption of hematopoietic and immune functions is a significant consequence of the long-term effects of radiation exposure. This study investigated the potential mechanisms by which ferulic acid (FA) acts as a radioprotective agent in mitigating radiation-induced immune damage. C57BL/6J mice were exposed to a dose of 6.0 Gy of ^60^Co γ irradiation. FA was administered at doses of 25, 50, and 100 mg/kg/d for 7 days before and 30 days following irradiation. We evaluated changes in peripheral blood cells, T and B lymphocytes, natural killer cells in the spleen, and hematopoietic stem/progenitor cells in the bone marrow (BM). Whole-genome transcriptome sequencing of BM was performed to explore potential mechanisms. FA administration resulted in a significant reduction in malonaldehyde levels (*p* < 0.0001), an increase in catalase and beta-nicotinamide adenine dinucleotide levels in serum (*p* < 0.05), and enhanced multipotent progenitors (*p* < 0.01) and common lymphoid progenitors (*p* < 0.05) in the BM. Additionally, there was an elevation in white blood cell levels, red blood cell levels, and hemoglobin levels in peripheral blood (*p* < 0.01). Transcriptome analysis indicated that FA reversed the radiation-induced expression of genes related to immunity and inflammation. Enzyme-linked immunosorbent assay experiments further demonstrated that FA reduced interleukin-6 levels in the BM and decreased JAK1, JAK2, and STAT3 protein content (*p* < 0.01). In conclusion, FA might mitigate hematopoietic and immune damage by modulating the JAK/STAT signaling pathway.

## 1. Introduction

Ionizing radiation (IR) poses significant risks to the human body once exposure surpasses a critical threshold. The primary danger linked to radiation is the production of reactive oxygen species (ROS), which lead to oxidative stress. A major consequence of prolonged radiation exposure is the impairment of hematopoietic and immune functions [1]. Acute exposure to IR can cause significant damage to bone marrow (BM) and peripheral immune cells, thereby reducing resistance to infections and hemorrhage and ultimately increasing mortality rates. Furthermore, radiation-induced tissue damage can trigger an inflammatory response, leading to the depletion of immune cell types critical for mitigating tissue injury. This, in turn, results in progressive and chronic tissue damage [2]. Radioprotective agents should promote the regeneration of damaged organs, modulate immune function, and alleviate oxidative stress. Currently, the primary radioprotective drugs in use or under investigation include aminothiols, nitroxide radicals, vitamins, hormones, and cytokines. However, many of these agents are associated with adverse side effects, such as vomiting and diarrhea. Natural active compounds, such as curcumin, genistein, and resveratrol, have demonstrated both IR-sensitizing effects on cancer cells and protective effects on normal cells [3]. In current scientific research, we have not yet developed a universally applicable pharmacological or supplementary approach to effectively defend against the generation of ROS. ROS are byproducts of cellular metabolism, and excessive ROS can lead to oxidative stress, damaging cellular structures and potentially causing various diseases. There is significant debate within the academic community about how to regulate and reduce ROS production using drugs or supplements. Firstly, the efficacy of different products in inhibiting ROS varies. For instance, widely studied antioxidants such as vitamin C, vitamin E, and coenzyme Q10 have produced inconsistent results across different studies. These discrepancies may arise from variations in experimental design, target populations, and specific health conditions. Secondly, dosage and regimen are also points of contention. Insufficient doses may fail to produce the desired antioxidant effects, while excessive doses could pose toxicity risks. For example, long-term high-dose consumption of certain antioxidants might interfere with normal cellular signaling processes and even increase the risk of certain diseases. Lastly, the potential side effects of antioxidants cannot be overlooked. Although some antioxidants show protective potential in the short term, their long-term safety has not been fully evaluated. Overconsumption of certain antioxidants, for instance, has been associated with an increased risk of cancer [4,5]. Ferulic acid (FA), a phenolic compound with the molecular structure of 4-hydroxy-3-methoxycinnamic acid (C10H10O4), is widely distributed in plants. FA exhibits a range of bioactive functions, serving not only as an antioxidant but also as an anti-inflammatory agent. It can eliminate excessive ROS and directly neutralize free radicals and free-radical-producing enzymes, thereby mitigating oxidative damage and reducing inflammatory responses [6]. Our previous studies have demonstrated that FA alleviates radiation-induced oxidative stress in vitro [7]. The radioprotective effect of FA has also been confirmed in vivo by other researchers [8,9]. In recent years, increasing evidence has shown that IR induces long-term BM damage primarily by promoting the senescence of hematopoietic stem cells (HSCs), which subsequently leads to immune senescence [10,11]. This study utilized a mouse model of hematopoietic and immune injury induced by 6.0 Gy whole-body IR. Utilizing transcriptomic analysis, combined with network pharmacology and molecular docking, the effect of FA on the Janus kinase (JAK)/signal transducer and activator of transcription (STAT) signaling pathway was investigated. The JAK/STAT signaling pathway plays a crucial role in mediating cellular responses to various cytokines and growth factors, thus influencing numerous biological processes such as proliferation, differentiation, and immune regulation. Dysregulation of this pathway has been implicated in a variety of diseases, including inflammatory disorders and cancers [12]. Given its pivotal role in these processes, our study focused on investigating specific endpoints related to the modulation of the JAK/STAT pathway to uncover potential therapeutic targets and improve our understanding of its contribution to mitigating radiation-induced HSC senescence and immune injury.

## 2. Results

### 2.1. Main Text

#### 2.1.1. FA Promoted the Recovery of Body Weight and Organ Index of Irradiated Mice

**Figure 1:**
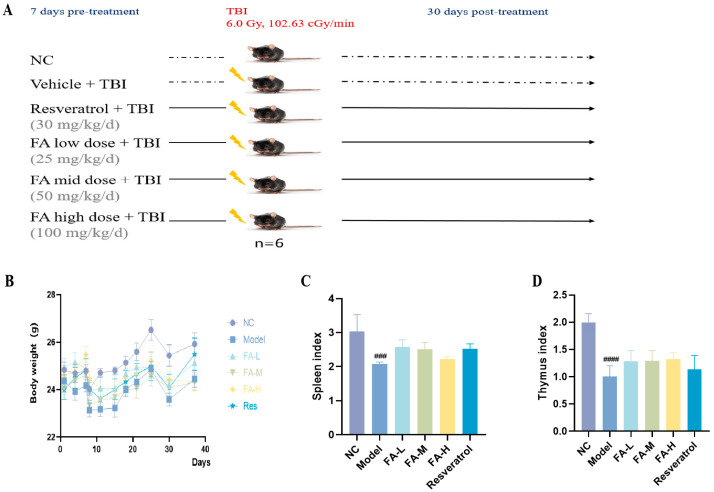
The effect of FA on the body weight and organ index of irradiated mice. (**A**) Flow diagram of experiment; (**B**) body weight; (**C**) spleen index; (**D**) thymus index. ^###^ *p* < 0.001, ^####^ *p* < 0.0001 vs. NC ($\bar{x}$ ± s, *n* = 6).

After irradiation, the body weight of the mice decreased, and the spleen and thymus indices were significantly reduced. Treatment with three different doses of FA partially restored the thymus index, while high doses of FA improved the spleen index (Figure 1B,C).

#### 2.1.2. FA Alleviated the Imbalance of Blood/Spleen Immunity in Irradiated Mice

**Figure 2:**
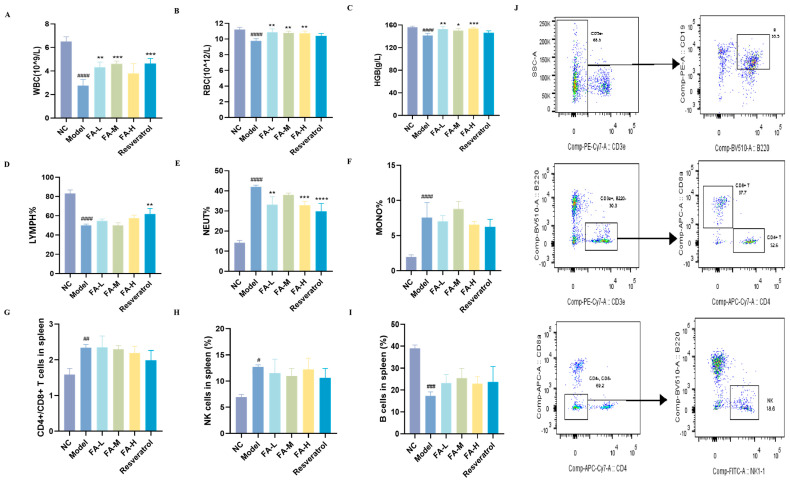
The effect of FA on the counts of blood cells and spleen immune cells in irradiated mice. The content of (**A**) WBCs; (**B**) RBCs; (**C**) HGB; (**D**) LYMPH%; (**E**) NEUT%; (**F**) MONO% in peripheral blood ((**A**–**F**), *n* = 4); (**G**) CD4+/CD8+ T cells; (**H**) NK cells; (**I**) B cells in the spleen ((**G**–**I**), *n* = 3); (**J**) flow cytometry gating strategy for splenic immune cells. * *p* < 0.05, ** *p* < 0.01, *** *p* < 0.001 vs. Model; ^#^ *p* < 0.05, ^##^ *p* < 0.01, ^###^ *p* < 0.001, ^####^ *p* < 0.0001 vs. NC ($\bar{x}$ ± s).

Irradiation led to decreased levels of white blood cells (WBCs), red blood cells (RBCs), hemoglobin (HGB), and the proportions of lymphocytes (LYMPH%) in the peripheral blood, as well as a reduction in B cells in the spleens of the mice (Figure 2A–D,I). Conversely, the proportions of neutrophils (NEUT%) and monocytes (MONO%), CD4+/CD8+ T cells, and natural killing (NK) cells increased (Figure 2E–H), indicating inflammation and compromised immune function. Treatment with FA mitigated these changes by increasing the levels of WBCs, RBCs, and HGB and decreasing NEUT%.

#### 2.1.3. FA Promoted the Recovery of Hematopoietic Stem and Progenitor Cells (HSPCs) in BM after Irradiation

**Figure 3:**
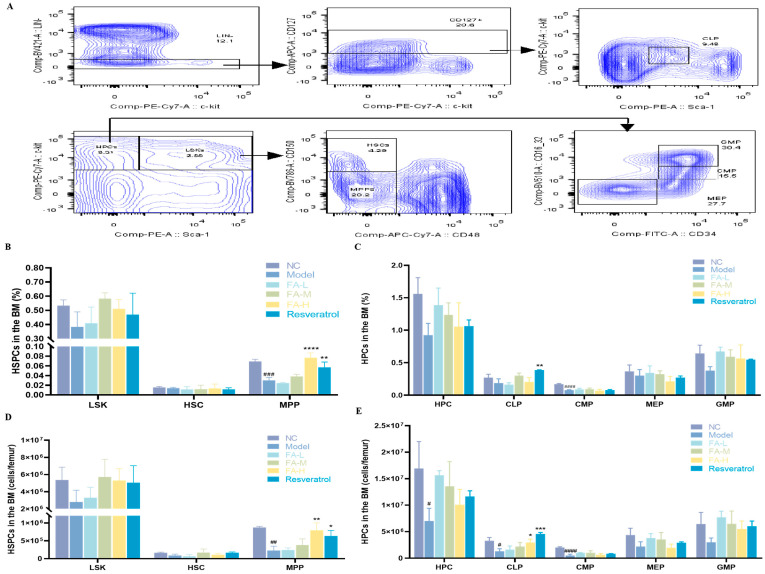
The effect of FA on the counts and proportion of HSPCs in BM following irradiation. (**A**) Flow cytometry gating strategy; the proportion of (**B**) LSK, HSC, and MPP; (**C**) HPC, CLP, CMP, megakaryocytic−erythroid progenitors (MEPs), and granulocyte−macrophage progenitors (GMPs); counts of (**D**) LSK, HSC, and MPP; (**E**) HPC, CLP, CMP, MEP, and GMP in BM. * *p* < 0.05, ** *p* < 0.01, *** *p* < 0.001, **** *p* < 0.0001 vs. Model; ^#^ *p* < 0.05, ^##^ *p* < 0.01, ^###^ *p* < 0.001, ^####^ *p* < 0.0001 versus NC ($\bar{x}$ ± s, *n* = 3).

Irradiated mice exhibited a decrease in the proportion and cell numbers of various populations of HSPCs, including a significant reduction in multipotent progenitors (MPPs), hematopoietic progenitors (HPCs), common lymphoid progenitors (CLPs), and common myeloid progenitors (CMPs) (Figure 3B–E). Treatment with 100 mg/kg FA or resveratrol markedly increased the levels of MPPs and CLPs.

#### 2.1.4. Network Pharmacology to Screen the Core Targets and Signaling Pathways Related to Aging in the Differential Genes

**Figure 4:**
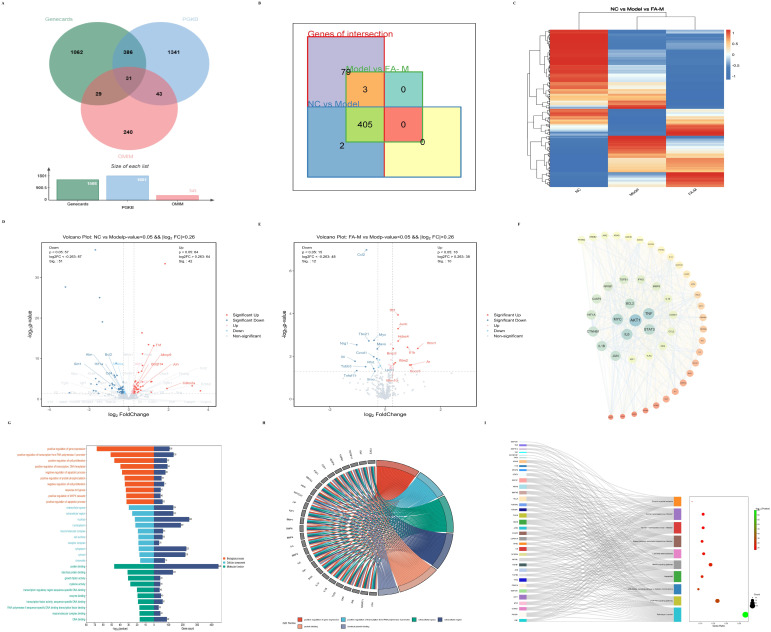
Core targets and signaling pathways related to aging identified from differentially expressed genes via network pharmacology. (**A**) Intersection of results obtained from the OMIM, GeneCards, and PGKB databases using “radiation induced age” and “radiation induced senescence” as search terms; (**B**) differentially expressed genes identified in the intersections; (**C**) heat map displaying differentially expressed genes across the three groups; Volcano plots of differential genes between (**D**) Model and NC; (**E**) FA-M and Model; (**F**) PPI network topology analysis, where the color gradient from blue to red and the font size from large to small indicate decreasing degree values; (**G**) GO enrichment analysis; (**H**) intersection genes from the GO enrichment analysis items with the smallest *p*-values; (**I**) targets involved in more than eight pathways in the Kyoto Encyclopedia of Genes and Genomes (KEGG) enrichment analysis.

The vast majority of radiation-induced aging-related genes were differentially expressed after irradiation and subsequent administration of treatment (Figure 4B–E). Further analysis of the commonly differentially expressed genes revealed that the core targets of the PPI network included AKT1, interleukin (IL) 6, STAT3, and TNF, among others (Figure 4F). As shown in Figure 4G, Gene Ontology (GO) enrichment analysis indicated significant involvement in gene expression, positive regulation of transcription from RNA polymerase II promoter, and other biological processes (BPs); protein binding and growth factor activity in molecular functions (MFs); and extracellular space, nucleus, and macromolecular complex in cellular components (CCs). Among the enriched genes, IL6, IL1β, TLR3, VEGF-α, and NOTCH1 were involved in BPs, MFs, and CCs simultaneously (Figure 4H). The core signaling pathways included pathways in cancer, the PI3K-Akt signaling pathway, and the AGE-RAGE signaling pathway in diabetic complications, involving genes such as AKT1, IL6, JAK2, and MAPK1 (Figure 4I).

#### 2.1.5. Molecular Docking Results of FA with JAK1 and JAK2

**Figure 5:**
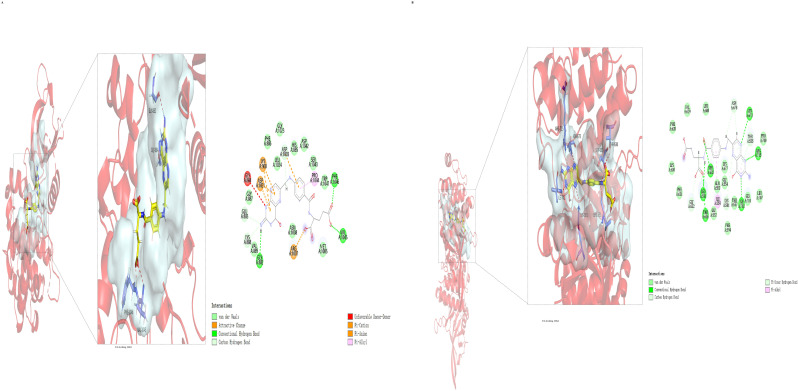
Molecular docking results of FA with JAK1 (**A**) and JAK2 (**B**). Molecular docking was performed using Discovery Studio 2023 with LibDock mode. Docking results were subsequently imported into PyMOL 2.3 for 3D visualization analysis.

Network pharmacology results suggest that FA may act on the JAK/STAT signaling pathway. This interaction was preliminarily verified through molecular docking of FA with JAK1 and JAK2 proteins (Figure 5, Table 1).

#### 2.1.6. FA Reduced Oxidative Stress and Inflammation in Serum and BM of Irradiated Mice

**Figure 6:**
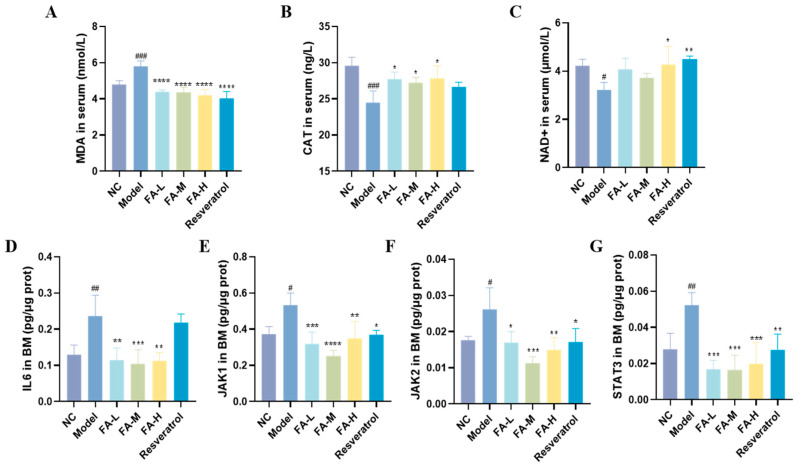
Expression of related proteins in mice serum and BM. The concentrations of (**A**) MDA, (**B**) CAT, and (**C**) NAD+ in serum are presented. Protein ratios (defined as the content of protein relative to the total protein concentration) for (**D**) IL6, (**E**) JAK1, (**F**) JAK2, and (**G**) STAT3 in BM are shown. * *p* < 0.05, ** *p* < 0.01, *** *p* < 0.001, **** *p* < 0.0001 vs. Model; ^#^ *p* < 0.05, ^##^ *p* < 0.01, ^###^ *p* < 0.001 vs. NC ($\bar{x}$ ± s, *n* = 4).

FA decreased serum malondialdehyde (MDA) concentration and increased catalase (CAT) and beta-nicotinamide adenine dinucleotide trihydrate (NAD+) levels in the irradiated mice (Figure 6A–C). Additionally, FA reduced the protein levels of IL6, JAK1, JAK2, and STAT3 in the BM of irradiated mice (Figure 6D–F). These results suggest that FA may act on the JAK/STAT signaling pathway, thereby reducing inflammation and oxidative stress in irradiated mice.

#### 2.1.7. FA Increased SIRT1 Gene and Decreased NOX4 Gene Expression in Spleen

**Figure 7:**
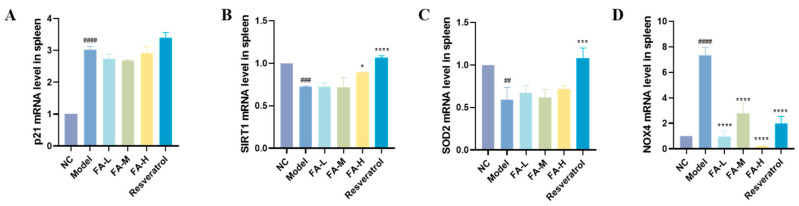
Expression levels of selected genes in mouse spleens. Expression levels of (**A**) p21, (**B**) SIRT1, (**C**) SOD2, and (**D**) NOX4 are shown. * *p* < 0.05, *** *p* < 0.001, **** *p* < 0.0001 vs. Model; ^##^ *p* < 0.01, ^###^ *p* < 0.001, ^####^ *p* < 0.0001 vs. NC ($\bar{x}$ ± s, *n* = 3).

SIRT1 plays an important role in the regulation of inflammation and cellular aging [13]. Its levels significantly decreased but could be increased by high-dose FA treatment (Figure 7B). Furthermore, the expression of the nicotinamide adenine dinucleotide phosphate oxidase 4 (NOX4) gene, a major source of ROS [14], was significantly reduced (Figure 7D). FA also influenced the expression of p21 and SOD2 genes.

#### 2.1.8. FA Reduced the Pathological Damage to the Spleen, Femur, and Thymus in Irradiated Mice

**Figure 8:**
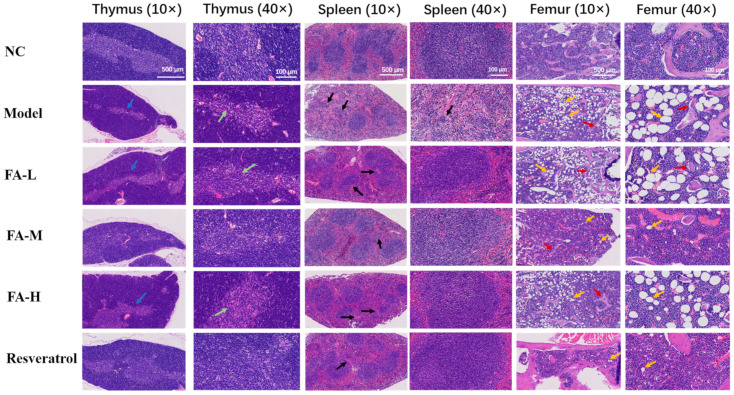
Results of H&E staining of the thymus, spleen, and femur in mice. Post-irradiation, a reduction in the thymus medulla was observed (blue arrow) along with a decreased number of thymic corpuscles and lymphocytes (green arrow). The splenic corpuscles were diminished, and the lymphoid nodules appeared smaller (black arrow). Additionally, there was a notable increase in adipocytes (yellow arrows) and a thinning of the bone trabeculae (red arrows).

In irradiated mice, the thymus exhibited marked atrophy, characterized by a significant reduction in the medulla, thymic bodies, and lymphocytes. The boundary between red pulp and white pulp in the spleen became indistinct, with a notable decrease in splenic bodies in the white pulp, a reduced volume of lymphoid nodules, and a decreased number of lymphocytes. In the femur, the number of adipocytes increased notably, and the bone trabeculae became thinner and decreased in number. These effects were improved after administration of the treatment (Figure 8).

#### 2.1.9. Other Possible Functions by Which FA Attenuated Immune Injury

**Figure 9:**
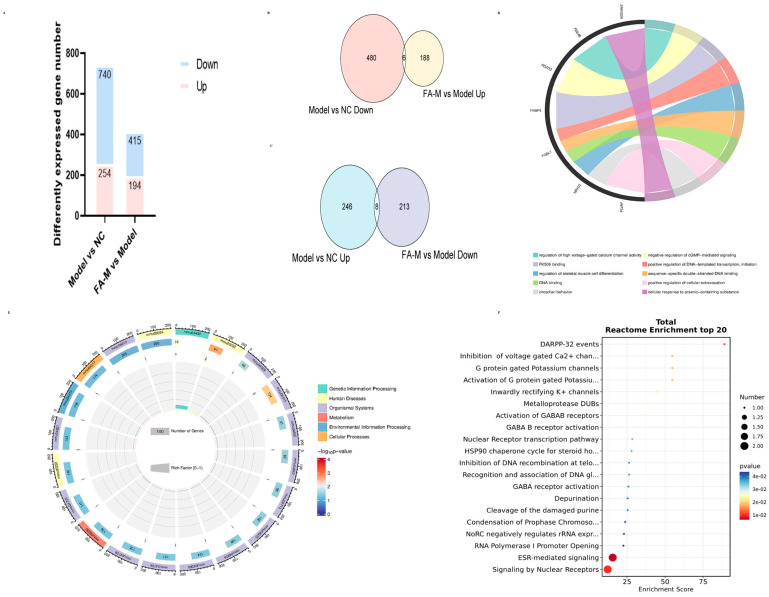
Further analysis of differentially expressed genes. (**A**) Statistics of differentially expressed genes; (**B**) Intersection of genes with significantly lower expression in the Model group and higher expression in the FA-M group. (**C**) Intersection of genes with significantly higher expression in the Model group and lower expression in the FA-M group. (**D**) Chord chart of GO enrichment analysis. (**E**) Circular diagram of KEGG enrichment analysis. (**F**) Bubble plot of Reactome Pathway enrichment analysis for intersection genes.

In addition to the differentially expressed genes related to aging mentioned above, other differentially expressed genes were further analyzed. Using the criteria of |Fold Change| ≥ 1.2, namely |log2FC| ≥ 0.26, and *p* < 0.05 as the variance analysis threshold [15], we identified 14 intersecting genes. These genes changed after irradiation but exhibited an opposite trend after administration. Specifically, the down-regulated genes were Fkbp5, Fosl1, H2ac19, Pdzd3, Plvap, Rusc2, Serpina3f, and Zfand2a, while the up-regulated genes were Dntt, Emp1, Kcnj5, Nr1d2, Pde4b, and Pknox2. Bioinformatic analysis was performed using the OECloud tools at https://cloud.oebiotech.com. The biological functions involved included regulation of skeletal muscle cell differentiation, negative regulation of cGMP-mediated signaling, and signaling by nuclear receptors, among others (Figure 9).

## 3. Discussion

Exposure to high doses of ionizing radiation results in progressive damage, manifesting as immunotoxicity, reproductive toxicity, and accelerated aging [16]. The primary contributors of this damage are ROS and chronic inflammation induced by cellular aging [17]. In this study, FA was selected to investigate its immunoprotective effects on irradiated mice due to its well-documented antioxidant and anti-inflammatory properties.

The results of the study demonstrated that irradiated mice experienced body weight loss, and both the spleen and thymus exhibited atrophy, as evidenced by organ indices and histopathological analysis. These effects were alleviated after FA administration. Peripheral blood cell counts provided insights into the immune and inflammatory processes occurring within the body [18]. The peripheral blood of mice in the Model group showed reductions in WBC count, RBC count, HGB levels, and LYMPH%, indicating impaired immune function [19]. Additionally, a marked increase in neutrophils and monocytes suggests the presence of inflammation [20]. MDA is a biomarker of lipid oxidative damage [21]. CAT is typically phosphorylated and activated by protein kinases to maintain hydrogen peroxide (H_2_O_2_) homeostasis, thereby protecting cells from oxidative stress [22]. NAD+, a coenzyme involved in redox reactions, is central to energy metabolism and can directly and indirectly influence many critical cellular functions [23]. In the Model group, serum MDA levels increased, while CAT and NAD+ levels decreased, further indicating the presence of inflammation. FA significantly increased the levels of WBCs, RBCs, and HGB, and it decreased LYMPH% in peripheral blood. FA also improved CAT and NAD+ levels while reducing MDA levels in serum, thereby alleviating the inflammatory damage in irradiated mice.

The increase in CD4+/CD8+ T cells and NK cells, along with the decrease in B cells in the spleen, indicates the presence of immune damage [24]. Further analysis revealed that the number of HSPCs in irradiated mice decreased, particularly MPPs, CMPs, and CLPs, which are the progenitors of RBCs, LYMPH, and T, B, and NK cells. Wang et al. demonstrated that whole-body ionizing radiation selectively induced the senescence of HSCs in mice and was characterized by a dramatic reduction in MPPs [25]. A high dose of FA had a significant effect on the recovery of MPPs and CLPs and showed some effect on the recovery of other progenitor groups, although significant variability was observed within the groups. These findings suggest that FA could mitigate the hematopoietic-immune damage observed in mice after 6 Gy whole-body irradiation, likely by alleviating radiation-induced HSC senescence.

We performed transcriptome sequencing of mouse BM to uncover additional insights. Genes related to radiation-induced senescence were identified through various databases. Among them, 405 genes were differentially expressed, suggesting that FA might alleviate radiation-induced senescence. Further analysis indicated that FA might exert its effects through the JAK/STAT signaling pathway. The JAK/STAT signaling pathway is central to extracellular cytokine receptor-mediated signal transduction, and it is involved in cell proliferation and differentiation, organ development, and immune homeostasis [26]. It is also essential for early hematopoiesis [27]. FA was found to reduce the levels of IL6, JAK1, JAK2, and STAT3 proteins in the BM microenvironment. Recent studies have shown that IR induces long-term BM damage primarily by inducing HSC senescence, which subsequently causes immune senescence [10,11]. Notably, Cdkn2a (p16) was significantly higher, and SIRT1 expression was lower in the Model group. p16 is a commonly used marker of cellular senescence [28], while the SIRT1 gene is often implicated in aging [29]. These results collectively suggest that FA could reduce inflammation in the BM microenvironment and alleviate radiation-induced HSC senescence, thereby improving hematopoietic and immune injury.

This study provides evidence that FA has the potential to mitigate the adverse effects of high-dose ionizing radiation, particularly in terms of immune and hematopoietic damage. However, there are several limitations. First, while our study demonstrated FA’s efficacy in mice, the translatability of these findings to humans remains to be validated. The mouse model, although informative, does not fully replicate the complexity of human physiology and immune responses. Second, the study primarily focused on the short-term effects of FA treatment; long-term outcomes and potential side effects require further exploration to ensure the comprehensive safety and efficacy of FA. Additionally, while the JAK/STAT pathway was identified as a potential mechanism, the study did not delve deeply into the pathway’s interactions with other cellular processes affected by radiation. Future research could expand on these findings by exploring combination therapies that might enhance FA’s protective effects, conducting long-term studies to assess sustained benefits and potential adverse effects and investigating other pathways that FA might influence to counteract radiation-induced damage. Such studies would not only bolster the understanding of FA’s protective mechanisms but also pave the way for developing more effective interventions against radiation-induced damage.

## 4. Materials and Methods

### 4.1. Reagents

Mouse CAT (MM-44125M1), MDA (MM-0897M1), NAD+ (MM-1010M1), IL-6 (MM-0163M1), JAK1 (MM-46967M1), JAK2 (MM-46942M1), and STAT3 (MM-45741M1) enzyme-linked immunosorbent assay (ELISA) kits were purchased from Jiangsu Meimian Industrial Co., Ltd. A bicinchoninic acid assay (BCA) protein quantitative kit (ZJ102) was obtained from Shanghai, China, Yaenzyme Biotechnology Co., Ltd. Hifair^®^ III 1st Strand cDNA Synthesis Super Mix for qPCR (11141ES60) and Hieff UNICON^®^ Universal Blue qPCR SYBR Master Mix (11141ES) were obtained from Yeasen Biotechnology (Shanghai, China) Co., Ltd. FA (S30464) and resveratrol (S30630), both with 98% purity, were purchased from Shanghai, China, Yuanye Bio-Technology Co., Ltd. Primers p21, SIRT1, SOD2, NOX4, and β-actin were synthesized in Beijing, China, Tianyi Huiyuan Biotechnology Co., Ltd. Antibodies used in flow cytometry are listed in Table 2. The sequences are shown in Table 3.

### 4.2. Animals and IR

Thirty-six SPF male C57BL/6J mice, weighing between 21 and 24 g, were purchased from Beijing Weitong Lihua Laboratory Animal Technology Co., Ltd., Beijing, China. The mice were housed at the Animal Center of the Academy of Military Medical Sciences (AMMS) and randomly divided (using computer-generated random numbers for group assignment) into six groups: a normal control group (NC); irradiation group (Model, vehicle+TBI); low-dose FA administration group (FA-L, 25 mg/kg/d i.g.+TBI); medium-dose FA administration group (FA-M, 50 mg/kg/d i.g.+TBI) [8]; high-dose FA administration group (FA-H, 100 mg/kg/d i.g.+TBI); and a positive drug resveratrol administration group (Resveratrol, 30 mg/kg/d i.g.+TBI) [30], with six mice in each group. After seven days of administration, all of the mice except those in the NC group were exposed to 6.0 Gy ^60^Co γ-ray at a dose rate of 102.63 cGy/min to induce hematopoietic and immune injury. Following irradiation, the mice continued to receive their respective treatments for an additional 30 days (Figure 1A). The experiment was conducted in accordance with the guidelines of the European Community and was approved by the Institutional Animal Care and Use Committee of AMMS: IACUC-DWZX-2023-547.

### 4.3. Blood Cell Counts

At the end of the administration period, blood samples were collected from the mice. Blood cell counts were determined using a hematology analyzer (Sysmex XN-1000™ Hematology Analyzer, Kobe, Japan). The counts included WBCs, RBCs, HGB, and NEUT%, LYMPH%, and MONO%.

### 4.4. Calculation of Organ Index

The mice in each group were weighed prior to euthanasia. Following euthanasia, the spleen and thymus tissues were removed and weighed. The organ indices were then calculated based on these weights.

Organ index = organ weight (mg)/body weight (g)

### 4.5. Flow Cytometry Analysis of HSPCs in BM and Immune Cells in Spleen

Single-cell samples from the BM and spleen were labeled with the appropriate fluorescent antibodies. The immunophenotypes of HSPCs, T cells, B cells, and NK cells in the spleen were then detected by flow cytometry [31].

### 4.6. Transcriptome Sequencing

Total RNA from BM samples of the NC, Model, and FA-M groups was extracted, enriched, fragmented, amplified, and finally sequenced by Shanghai OE Biotech Co., Ltd., Shanghai, China.

### 4.7. Network Pharmacology

Searches for “radiation induced aging” and “radiation induced senescence” were conducted in OMIM (Online Mendelian Inheritance in Man, https://www.omim.org), GeneCards (https://www.genecards.org), and PGKB (PharmGKB, https://www.pharmgkb.org). A Venn diagram was constructed using Bioinformatics tools to identify intersection genes. The differentially expressed genes from the intersection were introduced into STRING 11.5 (https://string-db.org) to construct a PPI (protein–protein interaction) network. Topological analysis of the PPI network was performed using Cytoscape 3.10.0 software. KEGG (Kyoto Encyclopedia of Genes and Genomes) and GO (Gene Ontology) enrichment analyses were conducted using DAVID (https://david.ncifcrf.gov).

### 4.8. Molecular Docking

According to the results of network pharmacology and transcriptome sequencing, the possible target protein structures were downloaded from the PDB database (https://www.rcsb.org). Molecular docking was performed using Discovery Studio 2023, with LibDock selected as the docking method. The docking results were then imported into PyMOL 2.3 for 3D visualization analysis.

### 4.9. Biochemical and Cytokine Analysis in Mouse Serum and BM

ELISA kits were used to detect the contents of MDA, CAT, and NAD+ in mouse serum, as well as the levels of IL-6, JAK1, JAK2, and STAT3 in BM. Protein in BM was quantified using the bicinchoninic acid (BCA) method. All operations were performed according to the manufacturer’s instructions.

### 4.10. Quantitative Real-Time PCR (qRT-PCR)

Total RNA was extracted from mouse spleens and reverse-transcribed into cDNA. Relative mRNA levels were quantified using the 2^−△△Ct^ method, with β-actin serving as an internal control. The primers used are listed in Table 3.

### 4.11. Hematoxylin–Eosin (H&E) Staining

The mouse thymus, spleen, and femur were fixed in 10% formalin, embedded in paraffin, and sectioned into 5 µm slices. H&E staining was performed for histopathological analysis.

### 4.12. Statistical Analyses

GraphPad Prism 8 was used to plot the data. One-way ANOVA was applied for statistical analysis between multiple groups, and a *t*-test was used for comparisons between two groups. A *p*-value of ≤0.05 was considered statistically significant.

## 5. Conclusions

FA exhibited a significant protective effect against IR-induced damage in mice, primarily through its antioxidant and anti-inflammatory properties. It improved peripheral blood cell counts and reduced markers of oxidative stress, such as MDA, while enhancing CAT and NAD+ levels. Additionally, FA facilitated the recovery of HSPCs, indicating its role in mitigating immune damage and cellular senescence induced by radiation. Transcriptome analysis further revealed that FA’s effects might be mediated through the JAK/STAT signaling pathway, which plays a crucial role in cytokine signaling, cell proliferation, and immune regulation. By modulating key proteins such as IL6, JAK1, JAK2, and STAT3, FA appeared to reduce inflammation and senescence markers in the BM microenvironment, suggesting its potential as a therapeutic agent for combating radiation-induced hematopoietic and immune injuries.

## Figures and Tables

**Table 1:** Molecular docking results of FA with JAK1 and JAK2.

Name	LibDock Score	Protein and Ligand Interaction
JAK1	139.437	Conventional Hydrogen Bond (PHE, VAL, GLY); Carbon Hydrogen Bond (ASP, GLU, LYS)
JAK2	157.567	Conventional Hydrogen Bond (ASN, ARG, SER, LEU, THR, ILE); Carbon Hydrogen Bond (ASN, THR, LYS, GLY)

**Table 2:** Antibodies used in flow cytometry.

Antibody	Clone	Conjugate	Source	LOT
Hematopoietic Lin cocktail	-	EF450	eBioscience	88-7772-72
CD117	ACK2	PE-Cy7	eBioscience	25-1172-82
LY-6/E	D7	PE	eBioscience	12-5981-82
CD34	RMA34	FITC	eBioscience	11-0341-85
CD127	A7R34	APC	eBioscience	17-1271-82
CD48	HM48-1	APC-EF780	eBioscience	47-0481-82
CD150	TC15-12F12.2	BV786	Biolegend	115937
CD16/32	S17011E	BV510	Biolegend	101333
CD19	HIB19	PE	eBioscience	12-0199-42
CD45R	RA3-6B2	EF506	eBioscience	69-0452-82
CD3e	145-2C11	PE-Cy7	eBioscience	25-0031-82
CD4	GK1.5	APC-EF780	eBioscience	47-0041-82
CD8a	53-6.7	APC	eBioscience	17-0081-82
NK1.1	PK136	FITC	eBioscience	11-5941-82
7-AAD	-	-	Biolegend	420404

**Table 3:** Primer sequences.

Gene	Forward Primer (5′-3′)	Reverse Primer (5′-3′)
β-actin	CCTCACTGTCCACCTTCCA	GGGTGTAAAACGCAGCTCA
p21	CCTGGTGATGTCCGACCTG	CCATGAGCGCATCGCAATC
SIRT1	TTGGCACCGATCCTCGAAC	CCCAGCTCCAGTCAGAACTAT
SOD2	ATTAACGCGCAGATCATGCA	TGTCCCCCACCATTGAACTT
NOX4	GATTTCTGGACCTTTGTGCCTTT	TGATGGTGACAGGTTTGTTGCT

## Data Availability

Data will be made available on request.
